# Ameliorative Effects of Zinc Oxide, in Either Conventional or Nanoformulation, Against Bisphenol A Toxicity on Reproductive Performance, Oxidative Status, Gene Expression and Histopathology in Adult Male Rats

**DOI:** 10.1007/s12011-023-03830-w

**Published:** 2023-09-08

**Authors:** Dina M. M. H. El-Kossi, Shawky S. Ibrahim, Kamel M. A. Hassanin, Nashwa Hamad, Noha A. Rashed, Ahmed Abdel-Wahab

**Affiliations:** 1 Physiology Department, Faculty of Veterinary Medicine, Minia University, El-Minia, 61519 Egypt; 2 Physiology Department, Faculty of Veterinary Medicine, Beni-Suef University, Beni-Suef, 62511 Egypt; 3 Biochemistry Department, Faculty of Veterinary Medicine, Minia University, El-Minia, 61519 Egypt; 4 Department of Pathology and Clinical Pathology, Faculty of Veterinary Medicine, Assiut University, Assiut, 71515 Egypt; 5 Department of Human Anatomy and Embryology, Faculty of Medicine, Assiut University, Assiut, 71515 Egypt

**Keywords:** Bisphenol A, Zinc, Zinc oxide nanoparticles, Testicular apoptosis

## Abstract

Bisphenol A (BPA) is a widely used endocrine disruptor that represents a significant risk to male reproductive function. Zinc (Zn) is vital for appropriate development of testes and to guarantee optimal testicular function and spermatogenesis. Our goal was to investigate if zinc oxide (ZnO), either in conventional or nanoformulation, could safeguard adult male rats’ reproductive performance against the damaging effects of BPA. Signaling expression of *CYP11A1* and *Nrf-2* in the testis, testicular oxidant-antioxidant status, *Bax/Bcl-2* apoptotic ratio, and histological examination of various reproductive organs were all evaluated. Twenty-eight adult male albino rats were divided randomly into 4 groups (7 animals each) including the control, BPA, conventional zinc oxide (cZnO) + BPA, and zinc oxide nanoparticles (ZnO-NPs) + BPA groups. The study was extended for 2 successive months. Our findings revealed strong negative effects of BPA on sperm cell characteristics such as sperm motility, viability, concentration and abnormalities. Additionally, BPA reduced serum levels of testosterone, triiodothyronine (T3), and thyroxine (T4). Also, it evoked marked oxidative stress in the testes; elevating malondialdehyde (MDA) and reducing total antioxidant capacity (TAC). BPA significantly downregulated testicular mRNA relative expression levels of *CYP11A1* and *Nrf-2*, compared to control. Testicular apoptosis was also prompted by increasing *Bax/ Bcl-2* ratio in testicular tissue. Histopathological findings in the testes, epididymis, prostate gland, and seminal vesicle confirmed the detrimental effects of BPA. Interestingly, cZnO and ZnO-NPs significantly alleviated all negative effects of BPA, but ZnO-NPs performed better. In conclusion, our findings point to ZnO, specifically ZnO-NPs, as a viable treatment for BPA-induced testicular dysfunction.

## Introduction

Endocrine-disrupting chemicals (EDCs) have drawn great attention because of their toxic and estrogenic activity as well as their proclivity to persist and bioaccumulate into body tissues [1]. Their risk stems from the fact that even at very low concentrations (less than 1 µg/L), they can have a variety of adverse effects [2]. Bisphenol A (BPA) is one of the most common EDCs that is widely ubiquitous in the environment. Annual production of BPA reached over 8 billion pounds and grew at a rate of 8%, with approximately 100 tons of BPA discharged into the environment each year [3]. BPA is frequently used in the polymerization of polycarbonate plastics, the design of thermal paper dyes, and numerous products that are regularly used by various livestock [4, 5]. Additionally, BPA is frequently used in the manufacture of epoxy resins, coating cans, and food packaging [6]. As a result, BPA exposure becomes extremely hazardous.

BPA was observed to impair sperm cell quality and reduced serum levels of testosterone and antioxidant enzymes [7]. Park and his colleagues also found that BPA caused Sertoli cell cytotoxicity. BPA downregulated *Bcl-2* expression (anti-apoptotic factor) and upregulated Caspase 3 and 9 and Bax (pro-apoptotic factors), the thing that pushes it to trigger male gametes apoptosis [8]. Moreover, low and high doses of BPA significantly reduced testicular volume, morphometry of seminiferous tubules and spermatogonial cells as well as the serum levels of testosterone and FSH [9]. Additionally, BPA is one of the estrogenic environmental pollutants, and at extremely low levels, it can bind to estrogenic receptors [10]. E-Screen (a cell proliferation assay based on the accelerated proliferation of human breast cancer cells “MCF-7” in the presence of estrogen-active chemicals) verified the estrogenic activity of several food contaminants, including BPA [11]. BPA’s estrogenic mimic action caused disturbance of folliculogenesis, elevation of CDKN2A relative expression, and induction of oxidative stress in female rats, elucidating the mechanism of BPA-induced polycystic ovarian syndrome (PCOS) [12].

Zinc (Zn) is required for normal development of testes and to guarantee optimal testicular function and spermatogenesis. Zinc is a common component of seminal plasma and it is indispensable for appropriate sperm cell maturation. Any variation in Zn levels had a significant negative impact on the testicular steroidogenic process [13]. Because of its higher Zn content and greater safety, conventional zinc oxide (cZnO) is preferred over other salts as a feed additive [14].

Nanotechnology has emerged as a viable technique for addressing a variety of medical challenges; Metallic nanoparticles, like Zn, have demonstrated remarkable protection against a number of medical assaults [15]. Furthermore, it has been noted that ZnO nanoparticles (ZnO-NPs) possess special features that set them apart from the conventional form in that they can freely cross a variety of bodily barriers, increasing the likelihood that target cells will receive a significant benefit from their effects [16]. Zinc oxide nanoparticles were found to provide protective effects against doxorubicin-induced testicular damage and genotoxicity based on their persuasive antioxidant and androgenic power [17]. In addition, ZnO-NPs significantly improved the testicular and epididymal histopathological changes that were caused by nicotine and restored the serum levels of testosterone, FSH and LH hormones to control levels [18].

The evidence for zinc oxide’s protective roles against EDCs-induced testicular dysfunction is still sketchy. As a result, we conducted this study to gain a thorough understanding of the mechanism by which ZnO, either conventional (cZnO) or nano (ZnO-NPs), could alleviate the potential negative effects of BPA in the testis of adult rats. We tracked the signaling expression of *CYP11A1* and *Nrf-2* in the testis, as well as the testicular oxidant-antioxidant status, the *Bax/Bcl-2* signaling apoptotic ratio, and histopathology of various reproductive organs.

## Materials and Methods

### Chemicals

Bisphenol A (BPA) was brought from Sigma-Aldrich Chemical Company in the USA and corn oil was used to dissolve BPA to bring a stock solution and the concentration was optimized to be 50 mg/kg BW/dose. The stock was kept in the refrigerator and could be used again in two days. Zinc oxide was purchased from Nasr Pharmaceutical Chemical Company, Egypt. Zinc oxide nanoparticles (ZnO-NPs), of particles size of 14-27.8 nm, were purchased from NanoTech Egypt for Photo-Electronics, Egypt. cZnO and ZnO-NPs were suspended in sterile distilled water to create the recommended dose (5 mg/kg BW) and mixed by vortex for 10 min before every administration. During the preparation of the cZnO and ZnO-NPs solution, safety precautions were taken, such as preventing skin contact with good-quality gloves and wearing a lab coat. In addition, the contaminated gloves were discarded in accordance with applicable legislation and good laboratory practices.

### Animals and Experimental Protocol

In this study, twenty-eight adult male albino rats were purchased from the house of laboratory animals in Assiut University, Egypt. The animals were 8 weeks old and weighed 133.26 ± 4.12 g BW on average. BPA-free cages (polypropylene cages) were used to house the animals. The animals were left for 2 weeks for acclimatization. A normal dark-light alternative cycle and a room temperature of 22 ± 2 °C were maintained during the experiment. *Ad libitum* strategy of drinking and feeding was established in this study using glass bottles and dishes rather than plastic ones to restrict the ingestion of BPA through the plastic equipment. The rats were fed a standard rat diet purchased from El-Wady Company, Egypt (24% protein, 7% crude fiber, 4% fat, 10% ash, and 55% carbohydrate “2490.00 Kcal/kg”). All experiments were under the guidance of the rules of the Institutional Review Board of Ethical Committee of Animal Care and Use in Scientific Research of Faculty of Veterinary Medicine, Minia University (IRB-FVM-MU) with approval number: IRB-FVM-MU -2023-102.

In the present study, the animals were randomly classified into four groups (7 rats each):

1. Control group: the animals were gavaged daily with 0.5 ml of vehicle (corn oil).
2. BPA group: The animals were gavaged with a daily dose of 50 mg BPA / kg BW according to WHO [19].
3. cZnO + BPA group: the animals received a daily dose of 50 mg BPA /kg BW by gavage plus 5 mg cZnO /kg BW intraperitoneally, twice/week according to Goma et al. [20].
4. ZnO-NPs + BPA group: the rats received the same daily dose of BPA plus 5 mg ZnO-NPs/Kg BW intraperitoneally, twice/week according to Goma et al. [20].

The experiment extended for 2 successive months that represent a complete spermatogenic cycle.

### Body Weight Gain and Gonado-somatic Index

Body weight gain (BWG) was computed for each animal by subtracting the final weight (at the end of the experiment) from initial one (at the beginning of the experiment). For gonado-somatic index (GSI), the following equation was followed:

$${\rm{GSI = }}\frac{{\rm The\,average\,testes\,weight}}{{\rm Final\,body\,weight}} \times 100$$

### Blood Sample Collection and Serum Separation for Biochemical Analysis

At the end of the experiment, the rats were anesthetized and blood samples were collected from the medial canthus of the eye. The collected samples were first left for 2 h. at room temperature and then centrifuged at 986 g for 20 min. to get the serum samples. The serum samples were then kept at -20 °C until be used for various biochemical analyses.

### Tissue Sampling

After blood sample collection, the rats were euthanized and their abdomen was opened to get different tissue samples for different purposes:

1. Testicular tissue portion was used for RT-qPCR analysis of the studied genes (*Cyp11a1, Nrf-2*, *Bax and Bcl-2)*. Thus, this portion was subjected to RNA isolation via TRI REAGENT® - RNA / DNA / PROTEIN ISOLATION REAGENT (Molecular Research Center, Inc. Cincinnati, OH., USA, Catalog number: TR 118) following the guidance of manufacturer’s protocol.
2. The second portion of testicular tissue was homogenized in phosphate buffer saline (PBS) to assess the oxidant-antioxidant markers.
3. The third portion of testis as well as specimens from epididymal, semen vesicular gland and prostatic tissue were all preserved in neutral buffered formalin (10%) for histological examination.

### Biochemical Assays

Measurement of serum testosterone levels was performed using ELISA kits (Cusabio, Wuhan, China; Catalog number: CSB-E05100r) according to the manufacturing protocol. Additionally the thyroid activity was assessed in this study by measuring serum levels of triiodothyronine (T3), and thyroxine (T4) using specific ELISA kits (Cusabio, Wuhan, China; Catalog numbers: CSB-E05085r and CSB-E05082r, respectively) as guided by the instructions of the manufacturer.

### Assessment of Oxidative Stress Parameters

Furthermore, the markers of oxidative stress including malondialdehyde (MDA) and total antioxidant capacity (TAC) were determined in the testicular tissue. For that, specific colorimetric test kits (Biodiagnostic company, Egypt and Catalog numbers: MD 25 29 and TA 25 13 for MDA and TAC, respectively) were used and performed as instructed by the manufacturer. MDA test based on a 30-minute reaction with thiobarbituric acid (TBA) in an acidic medium at 95 °C. This reaction resulted in the formation of a thiobarbituric acid reactive product. The resulting colored material absorbance was measured at 534 nm. TAC is determined by reacting the antioxidants in the sample with a predefined amount of exogenously provided hydrogen peroxide (H_2_O_2_). The antioxidants in the sample eliminated some of the H_2_O_2_, and the remaining amount of H_2_O_2_ was detected colorimetrically at 505 nm using an enzymatic reaction that converted 3, 5, dichloro-2-hydroxybenzensulphonate into a colored substance.

### Semen Characterization

Slicing of the tail of epididymis was done in a petri dish with PBS to get the semen sample. Sperm motility was observed using a light microscope (Olympus, CX31; Tokyo Japan). Sperm concentration was determined using a Neubauer chamber as outlined previously [21]. Sperm morphology and viability were evaluated using the eosin-nigrosine stain procedure as previously described [22].

### Real-Time Polymerase Chain Reaction (RT-PCR)

Reverse transcription, and qPCR assay of mRNAs of *Cyp11a1, Nrf-2*, *actin, Bax, Bcl-2 and GAPDH*’ in the testicular tissue were done by one step assay by using GoTaq® 1-Step RT-qPCR System kit (Promega Corporation, 2800 Woods Hollow Road, Madison, WI 53,711 USA; Catalog number: A6020) as instructed by the manufacturer. The relative quantification of mRNA expression was calculated using Biosystem Software integrated into the Applied Biosystems Real-Time PCR Instruments (Thermo fisher scientific, Waltham, MA USA). The primer sets of the studied genes were illustrated in Table 1.

The steps of thermal cycler programme included the following: (1) one cycle of reverse transcription (15 min at 37 ^o^C), (2) one cycle of RT inactivation/Hot-start activation (10 min at 95 ^o^C), (3) 40 cycles of qPCR including denaturation, annealing and extension, and (4) one cycle of extension, which is the last step (10 min, 72 ^o^C).

**Table 1:** Primer sets of the studied genes in the current study

	Sense	Antisense	Accession no
*CYP11A1*	5′- TCC GCT TTG CCT TGG AGT CTG TG-3′	5′- GAG GGT GAC GGC GTC GAT GAA-3′	NM_017286.3
*Nrf-2*	5′- ACG GTG GAG TTC AAT GAC-3′	5′- TGT TGG CTG TGC TTT AGG-3′	NM_031789.2
*β-actin*	5′- GGA GAT TAC TGC CCT GGC TCC TA-3′	5′- GAC TCA TCG TAC TCC TGC TTG CTG-3′	NM_031144.3
*Bax*	: 5′-AGC AAA CTG GTG CTC AAG GC-3′	5′-CCA CAA AGA TGG TCA CTG TC-3′	NM_017059
*Bcl-2*	5′-GTG GTG GAG GAA CTC TTC AG-3′	5′-GTT CCA CAA AGG CAT CCC AG-3′	NM_016993
*GAPDH*	5′- GTA TTG GGC GCC TGG TCA CC -3′	5′- CGC TCC TGG AAG ATG GTG ATG G -3′	NM_017008

### Histological Examination

In order to evaluate the histopathological alterations in the testes, epididymis, seminal vesicle, and prostate gland of rats of all groups, tissue specimens were freshly gathered. After fixation in neutral-buffered formalin 10%, the specimens were dehydrated in ascending concentrations of ethanol and embedded in paraffin. Thereafter, five-micron sections were prepared, mounted on glass slides, and stained with hematoxylin and eosin (H&E). Histopathological alterations were observed using a light microscope (Olympus, CX31; Tokyo Japan) and photographed using a digital camera (Toup view, LCMos10000KPA, China) [23]. The scoring of histopathological lesions was conducted by examining five random fields/section/organ. The lesions for each group were illustrated in a table to demonstrate their type, incidence, and severity [24].

### Statistical Analysis

The Statistical Package for Social Science (SPSS) software (SPSS for Windows, version 24, USA) was used to evaluate the data statistically. The data were analyzed for normal distribution by the Shapiro–Wilk test. The normally distributed data (BWG, GSI, sperm motility, sperm viability, sperm concentration, total sperm abnormalities, testosterone, T3, T4, TAC, MDA, *Nrf-2, Bax, Bcl-2 and Bax/Bcl-2* ratio) were examined by one-way analysis of variance (ANOVA) and post hoc Tukey’s test. Non-normally distributed data (*CYP11A1*), on the other hand, were evaluated using the Kruskal-Wallis test followed by a post hoc Mann-Whitney U test to determine which group produced the difference. The data expression was referred to as means ± standard error of the mean (SEM) for normally distributed continuous data and as median (min-max) for non-normally distributed continuous data, and p < 0.05 was considered to be significant.

## Results

### Characterization of ZnO-NPs

**Fig. 1:**
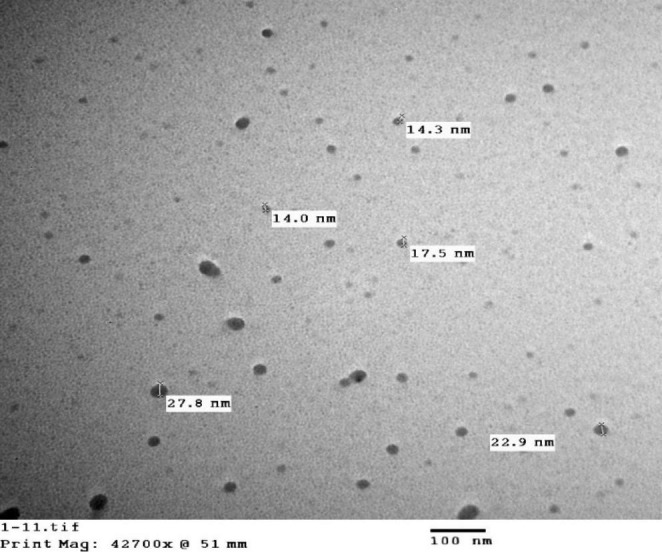
Transmission electron microscope (TEM) micrograph of ZnO-NPs illustrates spherical nanoparticles in the nano-range (14-27.8 nm) without any agglomeration

Zinc oxide nanoparticles were depicted in a TEM micrograph as spherical nanoparticles in the nanometer range (14–27.8 nm) without any agglomeration (Fig. 1).

### Body Weight Gain and Gonado-somatic Index

**Fig. 2:**
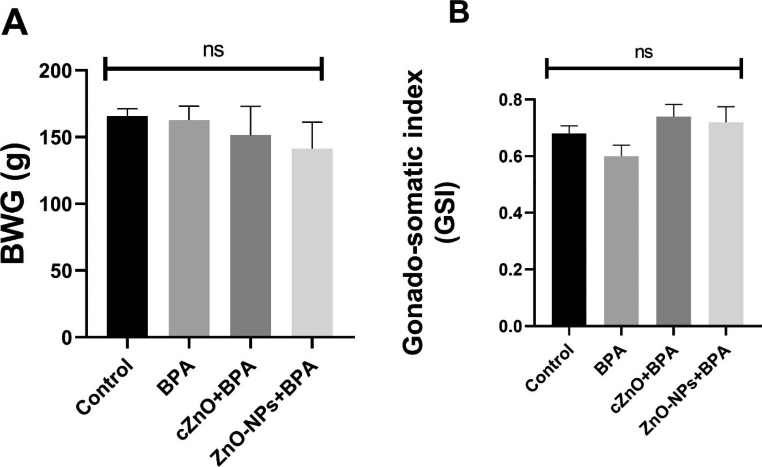
The effects of zinc oxide, in conventional or nanoparticle form, against bisphenol A toxicity on male rats’ body weight gain (**A**) and gonado-somatic index (**B**). BPA: bisphenol A, cZnO: conventional zinc oxide, ZnO-NPs: zinc oxide nanoparticles, BWG: body weight gain, GSI: gonado-somatic index. Data are expressed as mean ± standard error of the mean. ns: none-significant (*p* > 0.05)

The mean values of BWG (Fig. 2A) and GSI (Fig. 2B) in the cZnO and ZnO-NPs groups showed no significant differences when compared to the control or BPA groups. (*P* > 0.05).

### Sperm Cell Characteristics

**Fig. 3:**
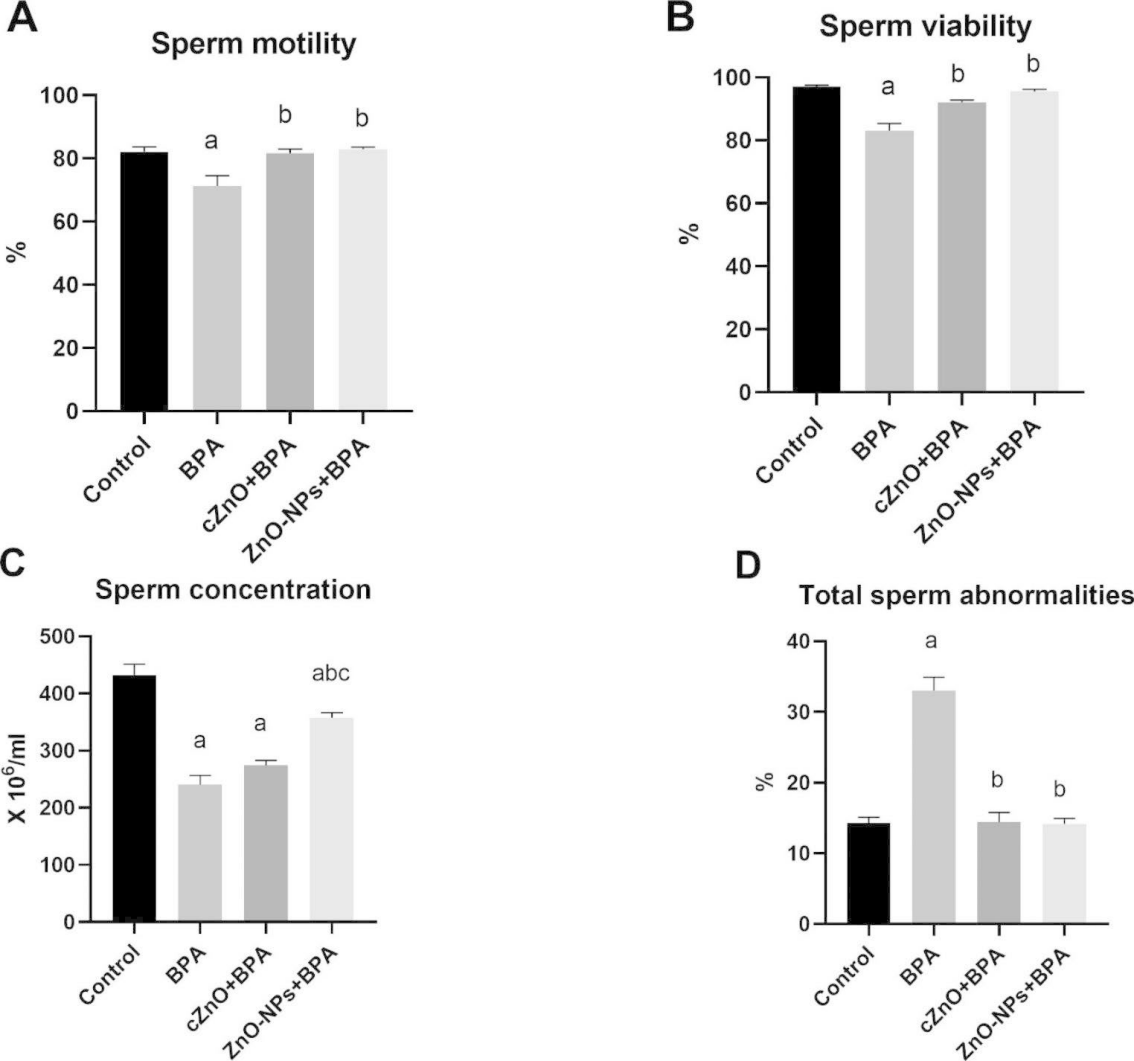
The effects of zinc oxide, in conventional or nanoparticle form, against bisphenol A toxicity on male rats’ semen characteristics. BPA: bisphenol A, cZnO: conventional zinc oxide, ZnO-NPs: zinc oxide nanoparticles. Data are expressed as mean ± standard error of the mean. ^a^*p* < 0.01 versus control group. ^b^*p* < 0.01 versus BPA group. ^c^*p* < 0.01 versus cZnO group

As shown in Fig. 3, BPA induced strong negative effects on sperm cell criteria. It dramatically reduced the percentages of sperm cell motility (Fig. 3A) and viability (Fig. 3B) as well as decreased sperm cell concentration (Fig. 3C) whereas the percentages of sperm cell abnormalities (Fig. 3D) were markedly increased in comparison to control group (*p* < 0.01). On the other side, cZnO and ZnO-NPs significantly improved BPA’s negative effects on all of the studied sperm cell parameters (*p* < 0.01).

### Levels of Hormones

Serum levels of testosterone, T3 and T4 showed a significant decrement (*p* < 0.001) in BPA group compared to the control (Table 2). cZnO and ZnO-NPs, on the contrary, significantly improved the serum levels of such hormones compared to BPA (*p* < 0.001). Additionally, ZnO-NPs performed better than cZnO (*p* < 0.001).

**Table 2:** The effects of zinc oxide, in conventional or nanoparticle form, against bisphenol A on serum hormonal assay and oxidative stress markers in adult male rats (Mean ± SE)

Group	Testosterone ng/dL	T3 ng/dL	T4 ng/dL	MDA (nmol /g testicular tissues)	TAC (mmol/ g testicular tissues)
Control	2.36 ± 0.055	1.91 ± 0.057	7.37 ± 0.110	0.87 ± 0.018	1.90 ± 0.029
BPA	0.89 ± 0.025^a^	0.72 ± 0.028 ^a^	2.75 ± 0.131 ^a^	2.89 ± 0.027 ^a^	0.66 ± 0.026 ^a^
cZnO + BPA	1.84 ± 0.027 ^ab^	1.51 ± 0.021^ab^	5.86 ± 0.091 ^ab^	1.91 ± 0.011 ^ab^	1.48 ± 0.015 ^ab^
ZnO-NPs + BPA	2.25 ± 0.015 ^bc^	1.83 ± 0.011 ^bc^	7.01 ± 0.040 ^bc^	1.62 ± 0.016 ^abc^	1.45 ± 0.016 ^ab^

SE: standard error, BPA: bisphenol A, cZnO: conventional zinc oxide, ZnO-NPs: zinc oxide nanoparticles, T3: triiodothyronine, T4: thyroxine, MDA: malondialdehyde, TAC: total antioxidant capacity

^a^*p* < 0.001 versus control group

^b^*p* < 0.001 versus BPA group

^c^*p* < 0.001 versus cZnO group

### Oxidative Stress Markers

Marked oxidative stress was observed in the testes of BPA-treated animals (Table 2) as MDA increased while TAC decreased in comparison to the control group (*p* < 0.001). Conversely, cZnO and ZnO-NPs did an excellent job to abate the oxidative stress caused by BPA by enriching the testis with TAC and eliminating MDA (*p* < 0.001). ZnO-NPs were more effective than cZnO at lowering MDA levels (*p* < 0.001).

### mRNA Relative Expression Levels of CYP11A1 and Nrf-2 in the Testis of Rats

**Fig. 4:**
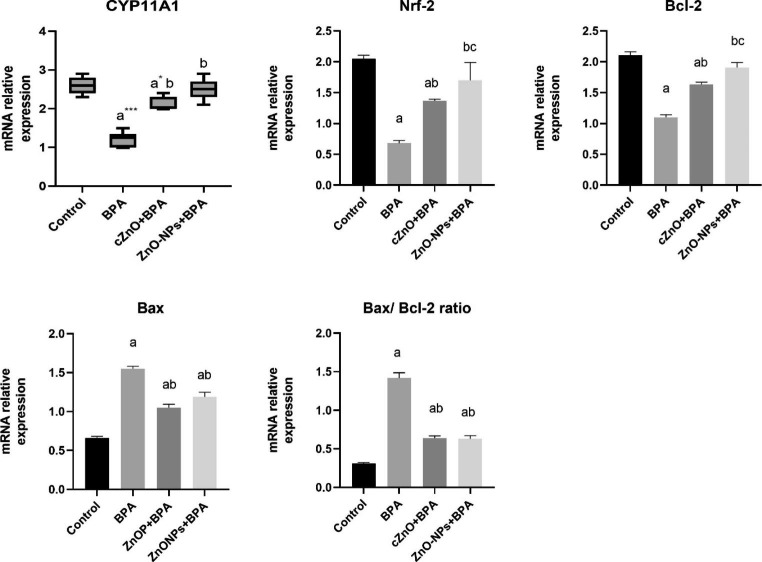
The effects of zinc oxide, in conventional or nanoparticle form, against bisphenol A on mRNA relative expression levels of *CYP11A1*, *Nrf-2*, *Bcl-2*, and *Bax* in the testis of rats. BPA: bisphenol A, cZnO: conventional zinc oxide, ZnO-NPs: zinc oxide nanoparticles. For *Nrf-2*, *Bcl-2*, *Bax, and Bax/ Bcl-2*ratio, comparisons were made by one-way analysis of variance (ANOVA) and post hoc Tukey’s test. Data are expressed as mean ± standard error of the mean. ^a^*p* < 0.001 versus control group, ^b^*p* < 0.001 versus BPA group, ^c^*p* < 0.001 versus cZnO group. For *CYP11A1*, comparisons were made using the Kruskal-Wallis test and the Mann-Whitney U test. Values are expressed as median (min-max). ^a***^p = 0.001 and ^a*^p = 0.002 versus control group, ^b^*p*=0.001 versus BPA group

BPA significantly downregulated testicular mRNA relative expression levels of *CYP11A1* (a marker of steroidogenesis) (*p* = 0.001) and *Nrf-2* (a marker of oxidative stress) (*p* < 0.001), relative to the control group (Fig. 4). It’s interesting to note that when BPA was combined with either cZnO or ZnO-NPs, the levels of the testicular mRNA transcripts for *CYP11A1* and *Nrf-2* were dramatically increased (*p* = 0.001 and *p* < 0.001, respectively). The act of ZnO-NPs was greater than cZnO in terms of its effect on testicular mRNA’s transcript level of *Nrf-2* (*p* < 0.001).

### mRNA Relative Expression Levels of Bcl-2 and Bax in the Testis of Rats as a Marker for Testicular Apoptosis

BPA significantly triggered testicular apoptosis via increasing Bax/ Bcl-2 ratio in the testicular tissue, relative to control group (*p* < 0.001) (Fig. 4). On the other hand, Bax/ Bcl-2 ratio was observed to be markedly recovered with either cZnO or ZnO-NPs in comparison to BPA (*p* < 0.001). Furthermore, the anti-apoptotic effects of cZnO and ZnO-NPs were observed to be comparable.

### Histopathological Findings

The histopathological alterations detected in the testes, epididymis, seminal vesicle, and prostate gland were illustrated in Table 3 which summarizes the incidence and severity of lesions observed in different experimental groups.

**Table 3:** Severity and incidence of histopathological lesions in testes, epididymis, seminal vesicle, and prostate gland of different groups

Lesion	Severity^a^ and Incidence^b^ of lesions in different groups			
	Control	BPA	cZnO + BPA	ZnO-NPs + BPA
*Testes*: Congestion	− (7)	+++ (7)	+ (7)	− (7)
Interstitial edema	− (7)	+++ (7)	++ (6)	− (7)
Degeneration of germinal epithelium	− (7)	+++(7)	++ (5)	− (7)
Intratubular giant cell formation	− (7)	+++ (7)	− (7)	− (7)
Leydig cell hyperplasia	− (7)	++ (7)	− (7)	− (7)
*Epididymis*: Vacuolation of germinal epithelium	− (7)	++ (7)	− (7)	− (7)
Sloughed germinal epithelium	− (7)	++ (5)	− (7)	− (7)
Hyperplasia of lining epithelium	− (7)	+ (3)	− (7)	− (7)
Intertubular inflammatory infiltration	− (7)	+++ (7)	+ (7)	− (7)
Absence of sperms in the lumina of epididymal tubules	− (7)	++ (4)	− (7)	
*Seminal vesicle*: Degeneration of the lining epithelium	− (7)	+++ (7)	− (7)	− (7)
Hyperplasia of lining epithelium	− (7)	+++ (3)	+ (2)	− (7)
Vacuolation in muscular wall	− (7)	+++ (7)	− (7)	− (7)
Congestion	− (7)	++(6)	− (7)	− (7)
Inflammatory infiltration	− (7)	+++(6)	− (7)	− (7)
*Prostate gland*: Glandular necrosis	− (7)	++ (6)	− (7)	− (7)
Glandular ectasia	− (7)	++ (7)	− (7)	− (7)
Interstitial edema	− (7)	+++ (7)	+ (7)	− (7)
Congestion	− (7)	+++ (7)	− (7)	− (7)

BPA: bisphenol A, cZnO: conventional zinc oxide, ZnO-NPs: zinc oxide nanoparticles

^a^ Lesion severity score: (−) absence of the lesion = 0%, (+) mild = 5–25%, (++) moderate = 26–50%, and (+++) severe ≥ 50% of the examined tissue sections

^b^ Number of rats with lesions per total examined (seven rats)

**Fig. 5:**
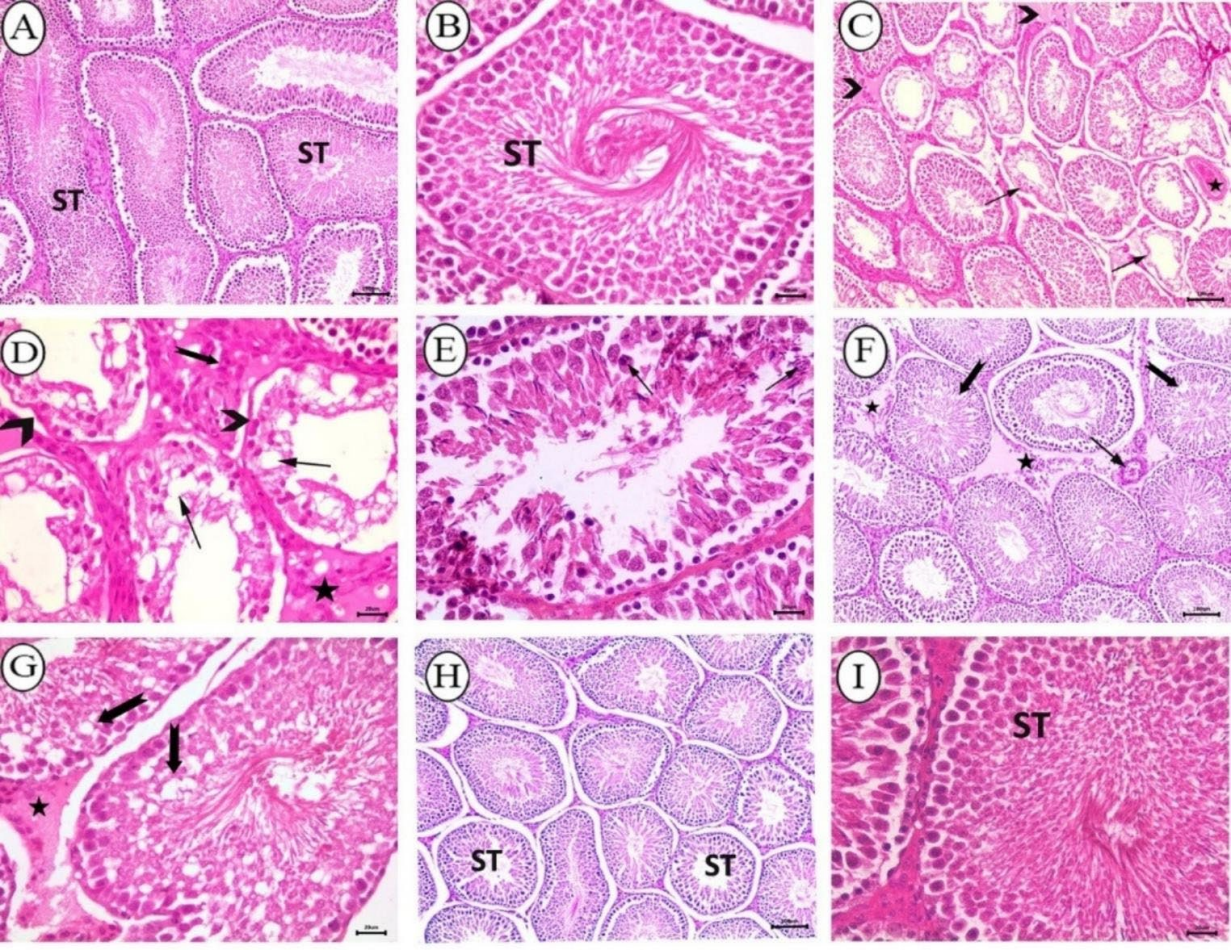
Histopathological analysis of testes from all groups stained with H&E. (**A-B**) Control group depicting normal appearance of seminiferous tubules (ST), and interstitial tissue, (A, bar = 100 μm; B, bar = 20 μm). (**C–E**) BPA-received rats; (**C**) depicting shrinked degenerated tubules (arrow), congestion of testicular blood vessel (star), and interstitial edema (arrow head), bar = 100; (**D**) depicting degenerated tubules lined with mono- or bi-layered vacuolated germ cells with no sperms in their lumina (arrow), undulant basement membrane (arrow head), leydig cells hyperplasia (notched arrow), and edema (star), bar = 20 μm, (**E**) depicting intratubular spermatogenic multinucleated giant cells (arrow), bar = 20 μm. (**F-G**) BPA + cZnO -received rats depicting vacuolation of germinal epithelium (notched arrow), interstitial edema admixed with small numbers of mononuclear cells (star), and vascular congestion (arrow), (F, bar = 100 μm; G, bar = 50 μm). (**H-I**) BPA + ZnO-NPs- received group depicting normal structural criteria of seminiferous tubules (ST) with improved spermatogenesis (H, bar = 100 μm; I, bar = 20 μm)

Hematoxylin and eosin-stained testicular sections of control rats showed normal appearance with no changes, neither in the seminiferous tubules nor interstitial tissue (Fig. 5A, B). However, sections from the BPA-received group had marked testicular degeneration, characterized by shrunk tubules lined with mono- or bi-layered vacuolated germinal epithelium with undulant basement membranes and no spermatozoa in the lumina. In addition, the testicular interstitium showed edema infiltrated with moderate numbers of lymphocytes, congestion of testicular blood vessels, and prominent hyperplasia of Leydig cells (Fig. 5C, D). Intratubular spermatogenic multinucleated giant cells were also evident in the examined sections of this group (Fig. 5E). In the testes of rats that received BPA + cZnO, the tubular structure and spermatogenesis were improved to some extent. There was vacuolation of germinal epithelium of small numbers of tubules, interstitial eosinophilic edema fluid admixed with small numbers of mononuclear cells, and slight vascular congestion (Fig. 5F, G). On the other hand, the rats in the group that received BPA + ZnO-NPs showed a significant enhancement in spermatogenesis, and the seminiferous tubules revealed normal structural criteria, indicating a notable improvement (Fig. 5H, I).

**Fig. 6:**
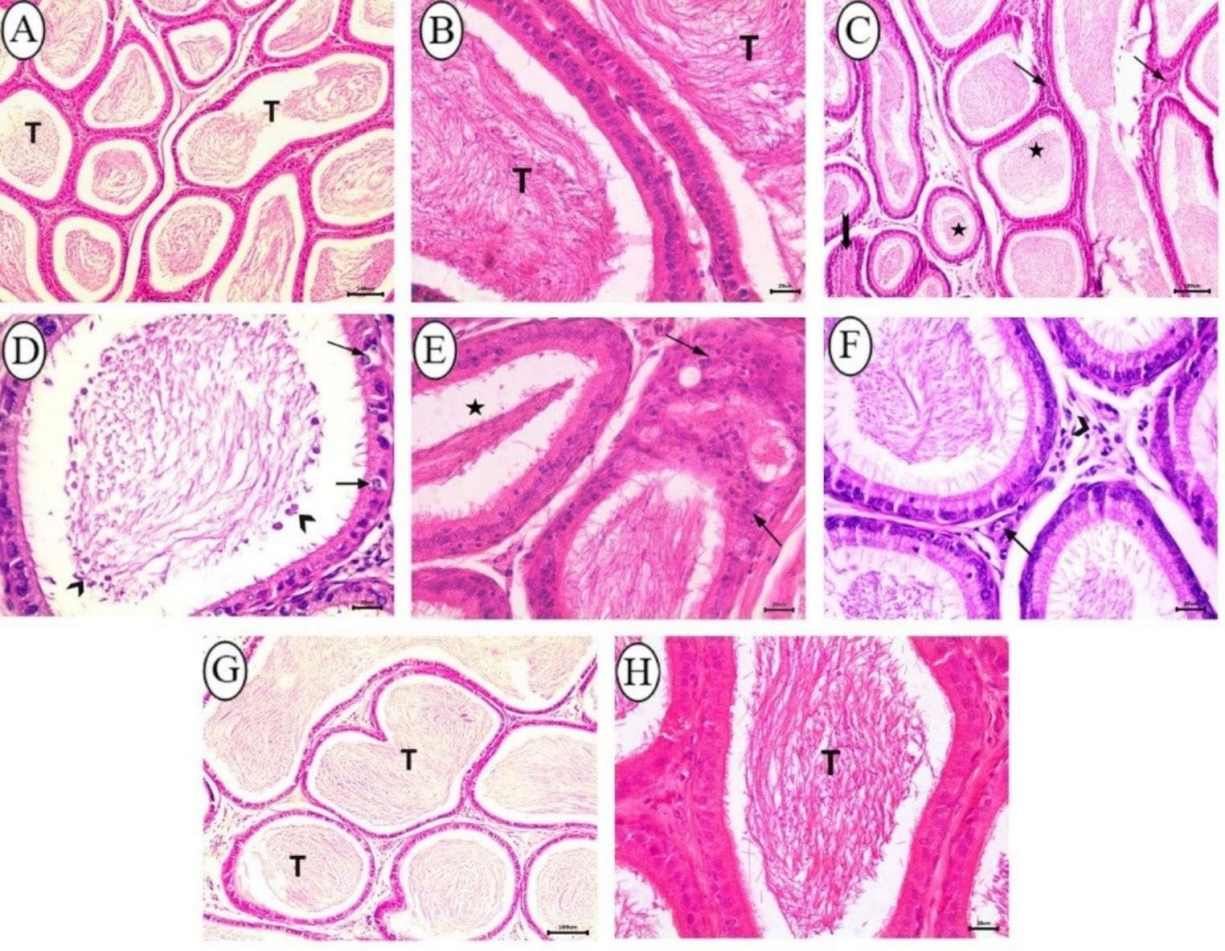
Histopathological analysis of epididymis from all groups stained with H&E. (**A-B**) Control group depicting normal architecture of epididymal tubules (T) lined with pseudostratifed columnar epithelium and normal sperm density, (A, bar = 100 μm; B, bar = 20 μm). (C–E) BPA-received rats; (**C**) depicting intertubular inflammatory cells infiltration (arrow), reduced sperm numbers (star), and complete absence of sperms inside the tubular lumen (notched arrow), bar = 100 μm; (**D**) depicting vacuolation of lining germ cells (arrow), and sloughed germ cells in lumen (arrow head), bar = 20 μm; (**E**) depicting hyperplasia of tubular lining cells (arrow) along with decreased sperm density (star), bar = 20 μm. (**F**) BPA + cZnO - received rats depicting slight interstitial congestion (arrow) and inflammatory infiltration (arrow head), bar = 20 μm. (**G-H**) BPA + ZnO-NPs - received group depicting normal appearance of epididymal tubular structure (T) and sperm content, (G, bar = 100 μm; H, bar = 20 μm)

The epididymis of rats in the control group displayed a normal structure of tubules lined with pseudostratified columnar epithelium and intertubular elements. The tubular lumina were densely filled with sperms (Fig. 6A, B). In the BPA-received group, the histopathological changes were remarkable and expressed by marked intertubular inflammatory infiltration, reduced mass of sperms inside the lumina of some tubules, and complete absence of luminal spermatozoa in other tubules (Fig. 6C). Moreover, germinal epithelium vacuolation and sloughed germ cells within lumens of convoluted tubules were detected in all examined sections of this group (Fig. 6D). Hyperplasia of lining epithelium accompanied by decreased number of sperms was observed in some epididymal ducts of three examined rats (Fig. 6E). In rats received BPA + cZnO, epididymal sections showed nearly normal architecture with slight intertubular vascular congestion and inflammatory infiltrate (Fig. 6F). Rats in the BPA + ZnO-NPs -received group demonstrated normal histological appearance along with normal sperm density (Fig. 6G, H).

**Fig. 7:**
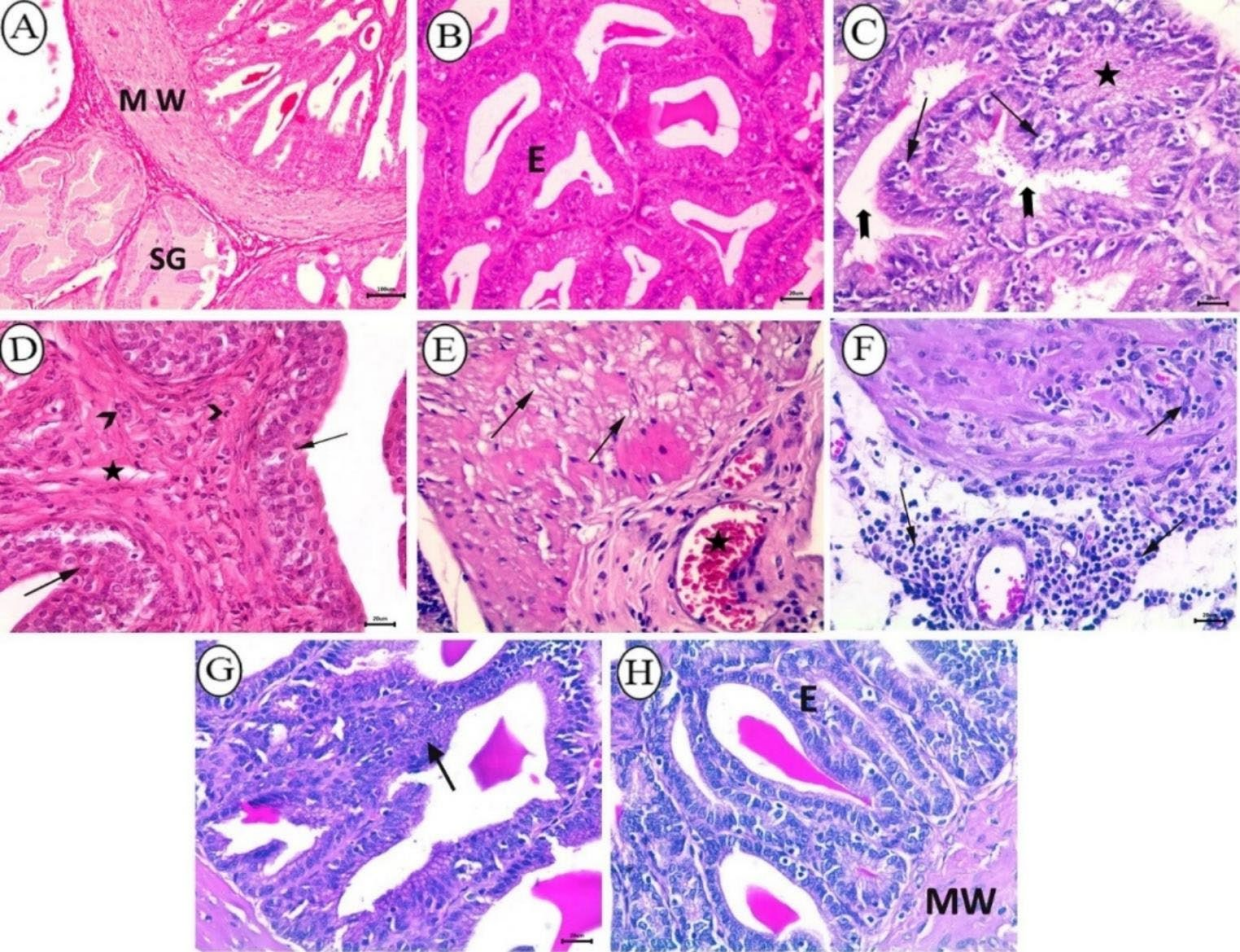
Histopathological analysis of seminal vesicle from all groups stained with H&E. (**A-B**) Control group depicting normal glands (SG) lined with pseudostratified tall columner epithelium (**E**) and outer muscular wall (MW) (A, bar = 100 μm; B, bar = 20 μm). (**C–F**) BPA-received rats; (**C**) depicting degenerative changes of lining epithelium (arrow), absence of luminal secretions (notched arrow), and eosinophilic debris in lumina of other glands (star), bar = 20 μm; (**D**) depicting hyperplasia of glandular epithelium (arrow) accompanied by proliferation of interstitial connective tissue (star) infiltrated with few neutrophils (arrow head), bar = 20 μm; (**E**) depicting cytoplasmic vacuolation of the muscular wall (arrow), and congested blood vessel (star), bar = 20 μm; (**F**) depicting infiltration of the muscular with aggregates of neutrophils and mononuclear cells (arrow), bar = 20 μm. (**G**) BPA + cZnO - received rats depicting focal epithelial hyperplasia (arrow), bar = 20 μm. (H) BPA + ZnO-NPs- received group depicting normal glandular epithelium (**E**) and surrounding muscular wall (MW), bar = 20 μm

Light microscopic examination of H&E stained tissue sections of the seminal vesicles of control rats showed normal glands lined with pseudostratified tall columnar epithelium and outer muscular wall (Fig. 7A, B). In contrast, BPA-treated rats showed marked degenerative changes in the lining epithelium. There was a complete absence of secretion within the lumina of most glands, whereas others contained eosinophilic debris (Fig. 7C). Prominent hyperplasia of the lining epithelium along with proliferation of connective tissue in lamina propria infiltrated with small numbers of neutrophils was observed in three rats from this group (Fig. 7D). The muscular layer exhibited vacuolation of cellular cytoplasm, congestion (Fig. 7E), and was infiltrated with aggregates of neutrophils and mononuclear cells (Fig. 7F). In group received BPA + cZnO, seminal glands healthy-looked in most examined sections, and focal hyperplasia of the lining epithelium was evident only in some examined ones (Fig. 7G). The rats which received BPA + ZnO-NPs showed normal glandular epithelium and surrounding muscle layer (Fig. 7H).

.

**Fig. 8:**
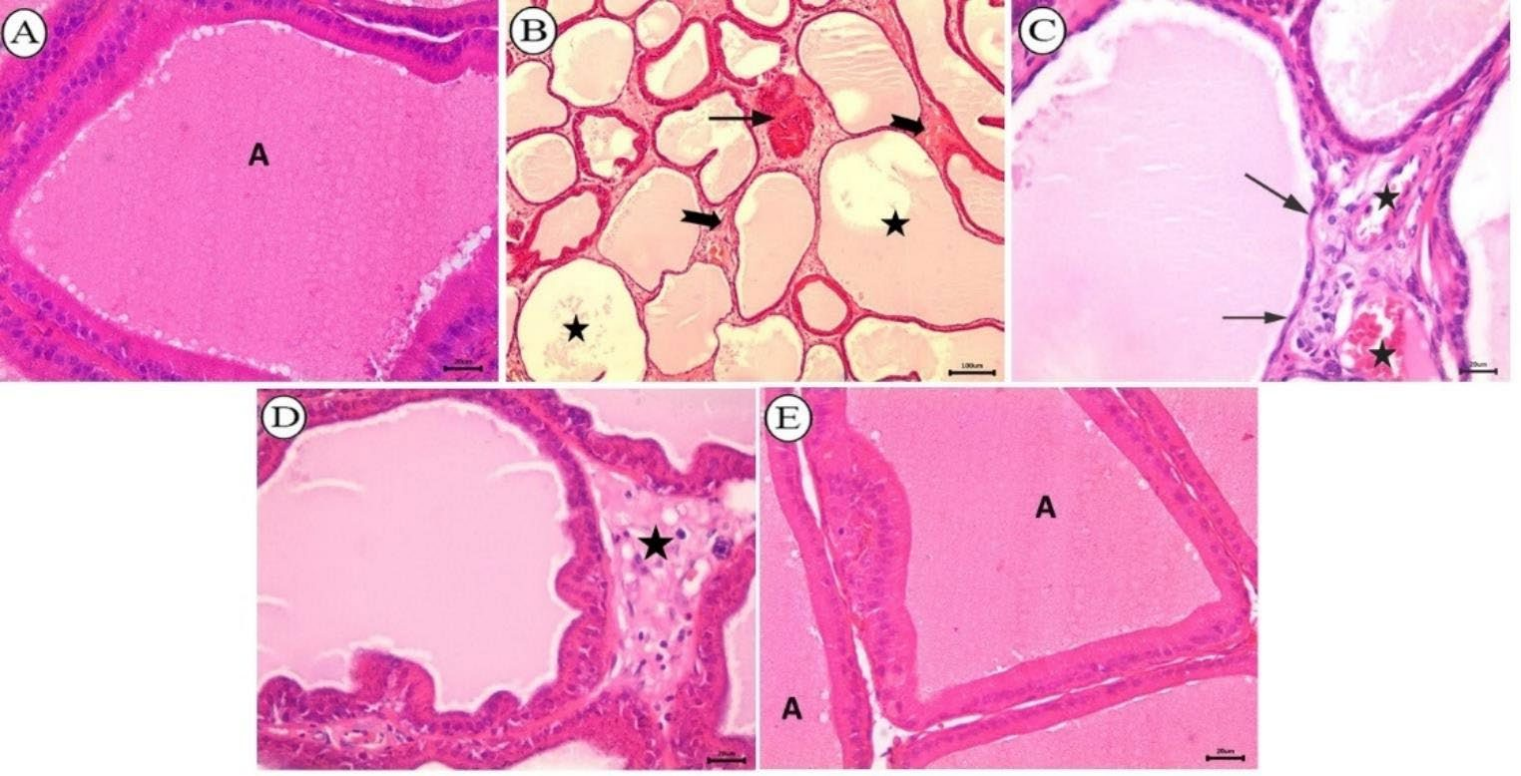
Histopathological analysis of prostate gland from all groups stained with H&E. (**A**) Control group depicting normal acinus (**A**) lined with tall columnar epithelium and contained luminal secretion, bar = = 20 μm. (**B–C**) BPA-received rats; (**B**) depicting ectatic acini (star), necrosed acini (arrow), and diffuse interstitial edema (notched arrow), bar = 100 μm; (**C**) depicting ectatic acini lined with attenuated epithelium (arrow), and congested blood vessels (star), bar = 20 μm. (D) BPA + cZnO- received rats depicting normal acinar structure with mild interstitial edema (star), bar = 20 μm. (**E**) BPA + ZnO-NPs- received group depicting normal acinar architecture (**A**), bar = 20 μm

After staining with H&E, a microscopic examination of prostate gland from the control group demonstrated normal acini lined with tall columnar epithelium and contained abundant luminal eosinophilic secretions (Fig. 8A). Conversely, the BPA-received group showed markedly ectatic acini lined with attenuated epithelial cells. Some acini were totally necrosed. Also, diffuse interstitial edema and congested blood vessels were detected (Fig. 8B, C). BPA + cZnO -received rats had normal acinar histomorphology with mild interstitial edema (Fig. 8D). Prostate sections from BPA + ZnO-NPs presented normal acinar architecture (Fig. 8E).

## Discussion

BPA is one of the most commonly investigated chemicals for its potential to cause endocrine disruption. BPA is widely disseminated in the environment and many commonplace applications, making it a serious risk to male fertility [5]. Therefore, the goal of the current study was to determine whether cZnO and ZnO-NPs could shield the testicular function of adult male rats against BPA toxicity.

Oxidative stress substantially compromises male fertility. This is because the testis is a relatively vulnerable organ to oxidative injury, owing to abundant cell division processes, higher levels of mitochondrial oxygen consumption within testes, and higher concentrations of unsaturated fatty acids [25]. Herein, BPA increased testicular levels of MDA and decreased those of TAC in comparison to the control group. These findings met an agreement with [26] that found a significant increment in MDA together with a significant decrement in antioxidant enzymes following BPA treatment for 45 days. Kaur et al. [27] also reported that ROS and lipid peroxidation levels were found to be significantly higher with BPA, while antioxidant levels (glutathione peroxidase) were significantly lower. In this regard, BPA has been demonstrated to trigger the formation of numerous radicals, including phenoxyl, hydroxyl, superoxide and peroxide radicals, following several enzymatic and non-enzymatic interactions [28]. These free radicals then caused lipid peroxidation and increased MDA, which is one of the most frequent lipoperoxidation products [29]. This phenomenon appears to be rationally tied to oxidative stress caused by BPA, which supports our findings.

In contrast, cZnO and ZnO-NPs did a good job of mitigating the oxidative stress generated by BPA by enriching the testis with TAC and eliminating MDA. Zn is an essential element that is the principal constituent of many metallic enzymes such as enzymatic antioxidants; it is also believed to weaken lipid peroxidation, which characterizes Zn’s anti-oxidative stress power [30]. Similar to our findings, ZnO-NPs were found to be efficient in decreasing MDA and increasing reduced glutathione in diabetic rats’ testes [31]. Moreover, treating rats with ZnO-NPs for 6 weeks succeeded to defend against aluminum (Al)-induced oxidative stress in the testes [32]. On the other side, Ziamajidi et al. [33] showed that ZnO-NPs at 200 mg/kg induced oxidative stress in the testes. We hypothesized that the authors’ high dose was responsible for this outcome, as it has been shown that at low concentrations, ZnO-NPs serve as antioxidants, while at high concentrations, they operate as pro-oxidants [20]. Furthermore, our findings indicated that ZnO-NPs were more effective than cZnO at lowering MDA levels. This could be justified by the study of Goma et al. [20] that found a significant increase of GSH and SOD testicular levels and a significant decrease of MDA with ZnO-NPs compared to the bulk form of ZnO, which could be related to the powerful penetrating activity of the nanoparticle form. Moreover, the in vitro study of Mohapatra et al. [34] highlighted that ZnO-NPs exhibited antioxidant activity stronger than the standard form, which confirmed our findings. Additionally, our findings from gene analyses showed that ZnO-NPs had a larger upregulating effect on the oxidative marker gene (Nrf-2) than cZnO, supporting our hypothesis.

Our data demonstrated that, whereas BPA had no detrimental effects on BWG or GSI, it had a significant negative effect on sperm cell quality. This is further corroborated by a significant reduction in serum testosterone levels, which is required for spermatogenesis. In line with our finding, it has been found that drinking water containing a low dose (50 µg/L) of BPS (BPA analog) for 70 days had no effect on GSI while significantly reducing sperm cell motility, viability, and concentration and increasing the percentage of sperm cell abnormalities [35]. BPS was also observed to inhibit steroidogenesis by interfering with the steroidogenic acute regulatory (StAR) protein. Also, Liu et al. [36] reported that BPA at low doses reduced sperm cell characteristics and serum testosterone levels. Incubating TM3 murine Leydig cells in vitro with BPA for two days reduced cell growth and viability and, as a result, testosterone production in a dose-dependent manner [37]. The detrimental effects of BPA on testosterone, and thus sperm quality, could be attributed to the oxidative stress provoked in the testes [35].

In this study, cZnO and ZnO-NPs both dramatically reduced BPA’s deleterious effects on testosterone production and sperm quality. Our data agreed with previous findings that observed favorable effects of ZnO-NPs on testosterone levels and sperm characteristics in diabetic rats [31]. Also, ZnO-NPs was effective candidate to counteract the negative effects of nicotine on testosterone and sperm quality [18]. When ZnO-NPs were added to a rooster’s semen extender that allowed for cooling storage, sperm quality, including motility, live-dead ratio, cell membrane stability, and oxidative stress, were significantly improved when compared to the control group, indicating the beneficial roles of ZnO-NPs in maintaining semen quality against the deteriorative effects of cooling storage [38]. It has been established that Zn is crucial for testosterone production due to its involvement in controlling the hypothalamic-pituitary axis and also because it inhibits aromatase activity, resulting in less conversion of more testosterone to estrogen [39]. Furthermore, Zn deficiency has been linked to a malfunction in the normal development of Leydig cells as well as impairment in LH receptors, both of which have a negative impact on testosterone biosynthesis [40]. In addition, the favorable effects of cZnO and ZnO-NPs on testosterone and sperm quality may potentially be attributable to their antioxidant activity, which protects testicular tissues from BPA-induced oxidative damage [32], which confirmed our findings. More notably, ZnO-NPs outperformed cZnO in terms of testosterone production, which could be attributed to ZnO-NPs’ stronger antioxidant activity than the conventional version [20].

It is widely established that thyroid hormones are mandatory for the normal function of the testis in the majority of species and perform crucial roles in the metabolism of sex hormones and spermatogenesis [41]. In the present study, there were negative effects of BPA on the serum levels of T3 and T4. Abdel-Wahab et al. [42] and Koutaki et al. [43] observed similar deleterious effects for BPA on thyroid gland. Chronic exposure to BPA has been linked to decreased thyroid-stimulating hormone receptor (TSHR) mRNA expression as well as detrimental effects on thyroid peroxidase, sodium-iodide co-transporter, and iodide pump activity [44]. These findings suggest another mechanism that may be contributing to the reduction in testosterone levels and impaired spermatogenesis caused by BPA. The activity of thyroid hormones was significantly recovered with either cZnO or ZnO-NPs. Zn was a promising treatment to protect thyroid gland against potassium dichromate thyrotoxicity [45]. Additionally, the beneficial effects on thyroid hormones may be due to the high antioxidant activity of cZnO or ZnO-NPs [32], which may protect the thyroid tissue from oxidative damage.

To understand the molecular mechanisms by which BPA impairs steroidogenesis and causes oxidative stress in the testes, we measured the testicular mRNA relative expression levels of the Cytochrome P450 Family 11 Subfamily A Member 1 (*CYP11A1*; a steroidogenesis marker) and nuclear factor (erythroid-derived 2)-like2 (*Nrf-2*; an oxidative stress marker). In this concern, *CYP11A1* gene is essential for testosterone production as it encodes the enzyme essential for conversion of cholesterol into pregnenolone which is an essential step for testosterone biosynthesis [46]. It is worth noting that alterations in *CYP11A1* expression were associated with reduced levels of steroids [47]. Concerning *Nrf-2*, it has been shown to be an effective oxidative stress biomarker in tissues [48]. Our data showed a significant reduction in the expression of *CYP11A1* and *Nrf-2* with BPA compared to control. In this regard, in vitro BPA exposure to mouse antral follicles downregulated *Cyp11a1* expression and also reduced testosterone levels on the fourth day of incubation [49]. When BPA was administered to mice via drinking water, the expression pattern of *CYP11A1* was reduced, resulting in low steroidogenesis activity [36]. The negative effects of BPA on *Nrf-2* have been reported by Mohammed et al. [44] that found significant alteration of *Nrf-2/HO-I* signaling pathway following exposure of rats to BPA for 5 weeks. As a result of these findings, we hypothesized that the main biological events by which BPA adversely affects testosterone production and spermatogenesis could be through downregulation of *CYP11A1* and *Nrf-2* expression in testicular tissues.

On the other side, the favorable effects of cZnO and ZnO-NPs on testosterone and sperm quality might be due to upregulating the *CYP11A1* and *Nrf-2* expression in testicular tissues. In this context, Daoud et al. [50] validated our findings and proved that ZnO-NPs can enhance the steroidogenic activity of testis against benzo[α] pyrene-induced testicular dysfunction through upregulation of testicular mRNA expression of *CYP11A1*. Moreover, the oxidative stress provoked in diabetic rats was significantly ameliorated by ZnO-NPs through upregulation of *Nrf-2* signaling pathway [51]. These findings supported our hypothesis and explained the molecular mechanism by which cZnO or ZnO-NPs can protect against BPA-induced steroidogenic machinery impairment and oxidative stress, hence alleviating the deleterious effects of BPA on testosterone production and spermatogenesis.

The induction of testicular apoptosis is another mechanism by which BPA may impair testicular function. The fundamental members that regulate the intrinsic pathway of apoptosis are *Bax and Bcl-2*. Apoptosis is induced if the *Bax/Bcl-2* ratio is not balanced [52]. In this study, BPA significantly increased the *Bax/Bcl-2* ratio in testicular tissue compared to the control group. In agreement with our findings, Zhang et al. [53] demonstrated an elevated *Bax/Bcl-2* ratio when goat Sertoli cells were exposed to BPA, resulting in apoptosis. As a result, we can speculate that BPA-induced testicular apoptosis is another factor contributing to the impairment of steroidogenesis and spermatogenesis.

In contrast to BPA, *the Bax/Bcl-2* ratio was found to be significantly recovered by cZnO and ZnO-NPs. The anti-apoptotic effects of ZnO-NPs were previously reported by Barakat et al. [54], who observed that ZnO-NPs had a substantial protective effect against cisplatin-induced renal tissue apoptosis. In that study, the authors found that ZnO-NPs downregulated *Bax* expression, which is consistent with our findings.

Our histopathological and morphometrically findings confirmed the detrimental effects of BPA on reproductive organs including testes, epididymis, seminal vesicles and prostate. These findings agreed with those of Zahra et al. [55], who observed damage in testicular tissue, including degeneration of seminiferous tubules and interstitial cells, as well as impairment of germ cells. All histopathological changes were demonstrated to improve slightly with cZnO and dramatically with ZnO-NPs, demonstrating their favorable effects, specifically the nanoparticle form, on testosterone production and sperm cell quality.

## Conclusion

It could be concluded that both cZnO and ZnO-NPs protect the male reproductive activity from BPA toxicity. They, specifically ZnO-NPs, significantly enhanced the quality of sperm cells, recovered the serum levels of testosterone, T3 and T4, relieved oxidative stress in the testes, upregulated the mRNA relative testicular expression levels of *CYP11A1* and *Nrf-2*, modulated *Bax/Bcl-2* and inhibited testicular apoptosis, and amended the histopathological changes caused by BPA. As a result, zinc oxide, specifically ZnO-NPs, could be regarded a viable therapy for BPA-induced male reproductive challenges.

## Data Availability

The data that support the findings of this study are available upon reasonable request.
